# Levofloxacin-Induced Toxic Epidermal Necrolysis

**DOI:** 10.7759/cureus.39504

**Published:** 2023-05-25

**Authors:** Andeebia A Khan, Urwah Aisha, Abdullah Hassan, Zaroon Haider, Daniel E Cook

**Affiliations:** 1 Internal Medicine, Fauji Foundation Hospital, Islamabad, PAK; 2 Medicine and Surgery, Nishtar Hospital Multan, Multan, PAK; 3 Internal Medicine, Mayo Hospital Lahore, Lahore, PAK; 4 Internal Medicine, Combined Military Hospital (CMH) Lahore Medical And Dental College, Lahore, PAK; 5 International Medical, Avalon University School of Medicine, Youngstown, USA

**Keywords:** toxic epidermal necrolysis (ten), toxicity, pathology, levofloxacin, dermatology

## Abstract

Toxic epidermal necrolysis (TEN), also known as Lyell's syndrome, is a severe episodic mucocutaneous reaction that is usually brought on by oral medications and/or sporadically by infections.

We report a case of a 19-year-old male with the presenting complaint of generalized skin blistering over the previous seven days at the dermatology outpatient clinic. The patient has had epilepsy since he was 10 years old. Due to an upper respiratory tract illness, a local healthcare facility recommended oral levofloxacin to him seven days ago. Levofloxacin-induced toxic epidermal necrolysis (TEN) was suspected based on the patient's medical history, physical examination, and research. On the basis of histological investigations and clinical correlation, the diagnosis of TEN was determined.

The mainstay of treatment after diagnosis was made was supportive care. The best methods for treating TEN involve stopping any potential causal agents and providing supportive care. The patient received care in the intensive care unit.

## Introduction

A severe episodic mucocutaneous reaction called toxic epidermal necrolysis (TEN), often known as Lyell's syndrome, is frequently brought on by medicines taken orally and/or infrequently by infections. With a mortality rate of 30-35%, TEN manifests as sheets of erythema, necrosis, and bullous epidermal detachment [1]. Since the discovery of the prototype chemical nalidixic acid in 1962, fluoroquinolones (FQs) are an important class of medications that are frequently administered for a variety of clinical problems [2]. They are divided into first- to fourth-generation FQs based on the molecular characteristics and the range of the antibacterial action. They have a favorable safety profile and are generally well-tolerated by patients [3]. However, reports of adverse drug reactions (ADR) after taking levofloxacin have been made. These ADRs have included everything from an acute purpuric reaction to idiosyncratic hepatotoxicity to toxic epidermal necrolysis (TEN) and Stevens-Johnson syndrome (SJS), both of which can be fatal [4].

One proposed mechanism for the development of TEN involves an immunological pathway wherein drug-modified epidermal cells are targeted. CD8 T-lymphocytes play an early role, and higher levels of soluble interleukin-2 receptors (sIL-2R) are found in blister fluid. Microscopically, eosinophilic epidermal necrosis and sub-epidermal bullae formation are observed. Dermal vessels show endothelial swelling, while basal and lower spinous layers of the epidermis exhibit structural damage with lamina densa clefts. Immuno-fluorescence tests are consistently negative.

## Case presentation

A 19-year-old male patient presented to the dermatology outpatient department with a complaint of generalized blistering of skin for the last seven days. The patient was a known case of epilepsy from 10 years of age. He was suggested oral levofloxacin by a local health care unit seven days back because of an upper respiratory tract infection. The patient was unable to produce any documented data regarding the ailment.

On the second day after starting the levofloxacin, the patient developed erythematous maculopapular rash. The rash started in the chest region. It progressed overnight on the face region, arms, and legs. The rash was tender and pruritic. There was a history of a burning sensation in the rash as well. However, the patient continued the medication during this interval.

On the fourth day, there was generalized blister formation and sloughing of the skin. Blisters were flaccid, contained clear fluid, and were diffusely scattered over the body. The patient further added that there was a burning sensation in the mouth as well. No ocular involvement was reported; however, there was a complaint of burning micturition along with other symptoms.

The patient was on a regulated dose of carbamazepine for the last nine years for his epilepsy. Epilepsy was in a controlled state, and no history of any skin lesion associated with anti-epileptic drug intake was found. Moreover, there was no history of photosensitivity, joint pain, weight loss, and night sweats.

**Figure 1:**
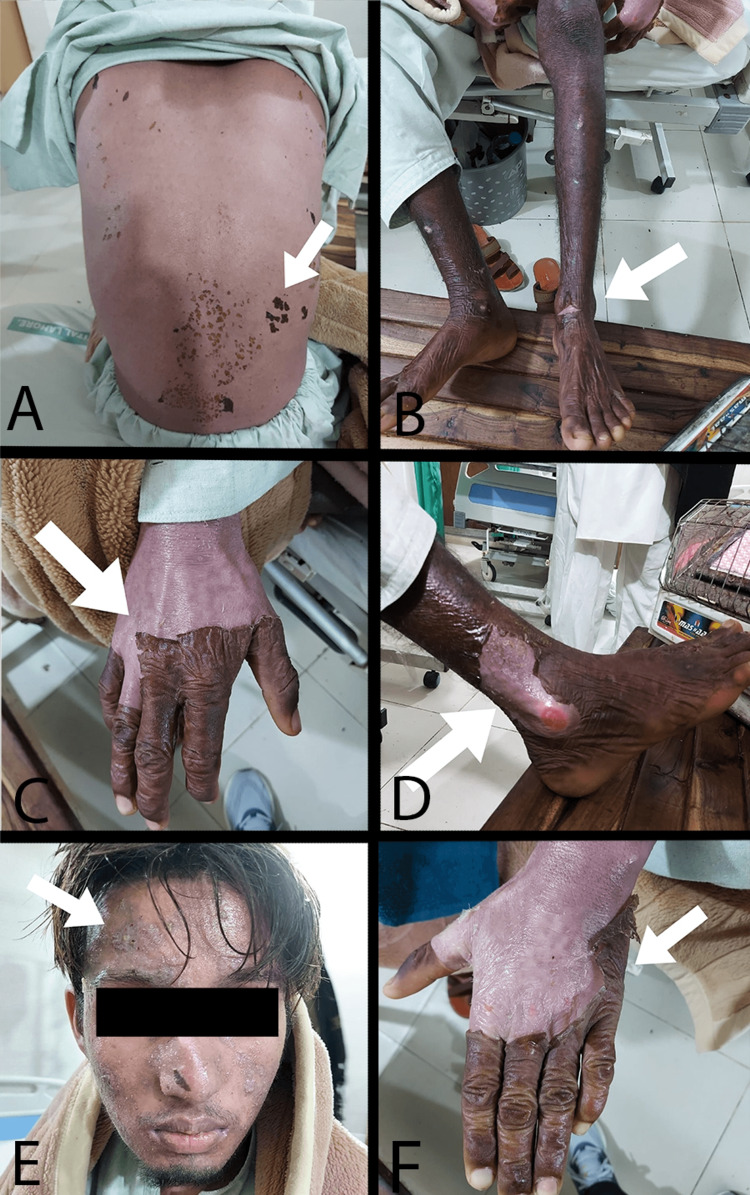
Toxic epidermal necrolysis with different skin features Sloughing of the epidermis can be seen on the left leg (B), right hand (C), right foot (D), and left hand (F). A toxic epidermal necrolysis ulcer is shown on the forehead (E). Whole back involvement is shown in A.

On examination, generalized erythema, large flaccid clear bullae, erosions on different parts of the body, and oral erosions were found. Nikolsky's sign was positive, which is the shearing away of skin at the edge of a lesion by the application of lateral pressure. Sloughing of the epidermis from the left leg, right hand, right foot, and left hand is shown in Figures 1B-D and 1F, respectively. A toxic epidermal necrolysis ulcer is shown on the forehead in Figure 1E. Whole back involvement is shown in Figure 1A.

For investigations, a complete blood count (CBC), liver function tests (LFTs), renal function tests (RFTs), coagulation profile, serum electrolytes, blood culture and urine complete examination was performed. All these features have been described in Table 1.

**Table 1:** Blood workup of the patient showing deranged findings INR - international normalized ratio, APTT - activated partial thromboplastin time, WBC count - white blood cell count, RBC - red blood cells, HCT - hematocrit, MCV - mean corpuscular volume, MCH - mean corpuscular hemoglobin, MCHC - mean corpuscular hemoglobin concentration, ALT - alanine transaminase, AST - aspartate aminotransferase, ALP - alkaline phosphatase

Coagulation profile	Value	Reference range
Prothrombin time - control	13	10-14 seconds
Prothrombin time - patient	14	Up to 13 seconds
INR	1.3	0.9-1.3
Control time	28	25-35 seconds
APTT	28	Up to 31 seconds
Hemogram		
WBC count	12.3	4-11 x10^9^/L
Total RBC	4.07	3.8-5.2 x10^12^/l
Hemoglobin	11.6	13-18 (g/dL)
HCT	38.1	35-46%
MCV	89.9	77-95 fl
MCH	27.4	26-32 (pg)
MCHC	30.4	32-36 (g/dL)
Platelets	445	150-400 x10^9^/L
Neutrophils	87	40-80%
Lymphocytes	12.4	20-40%
Monocytes	6	2-10%
Eosinophils	1	1-6%
Renal function tests		
Urea	23.24	10-50 mg/dl
Serum creatinine	0.55	0.5-0.9 mg/dl
Liver function tests		
Bilirubin total	0.4	0.3-1.2 mg/dl
Total protein	6.1	5.7-8.2 g/dl
Albumin	4	3.2-4.8 g/dl
ALT	420	Up to 40 U/L
AST	136	Up to 40 U/L
ALP	384	40-120 U/L
Serum electrolytes		
Sodium	134	135-145 mmol/L
Potassium	4.63	3.5-5 mmol/L
Chloride	99.9	98-107 mmol/L
Calcium	8.6	8.5-10.5 mg/dl
Inflammatory markers		
C-reactive protein	17	<10 mg/L

Initially, liver enzymes were found deranged, but the cause was attributed to levofloxacin intake. There was not any history of alcohol intake. The patient didn't have a history of diabetes mellitus. Hepatitis serologies were negative for any type of hepatitis. Ultrasound of the liver and abdomen did not give any positive findings; hence gallbladder pathologies were also eliminated. Thus, all the other probable causes of liver damage were ruled out.

On the basis of the Severity-of-Illness Score for Toxic Epidermal Necrolysis (SCORTEN), a score of one was allotted to the patient. This is because the compromised body surface was more than 10%. The mortality rate at a score of one is very low. It is equivalent to 3.2%, with a confidence interval of 0.1-16.7.

**Figure 2:**
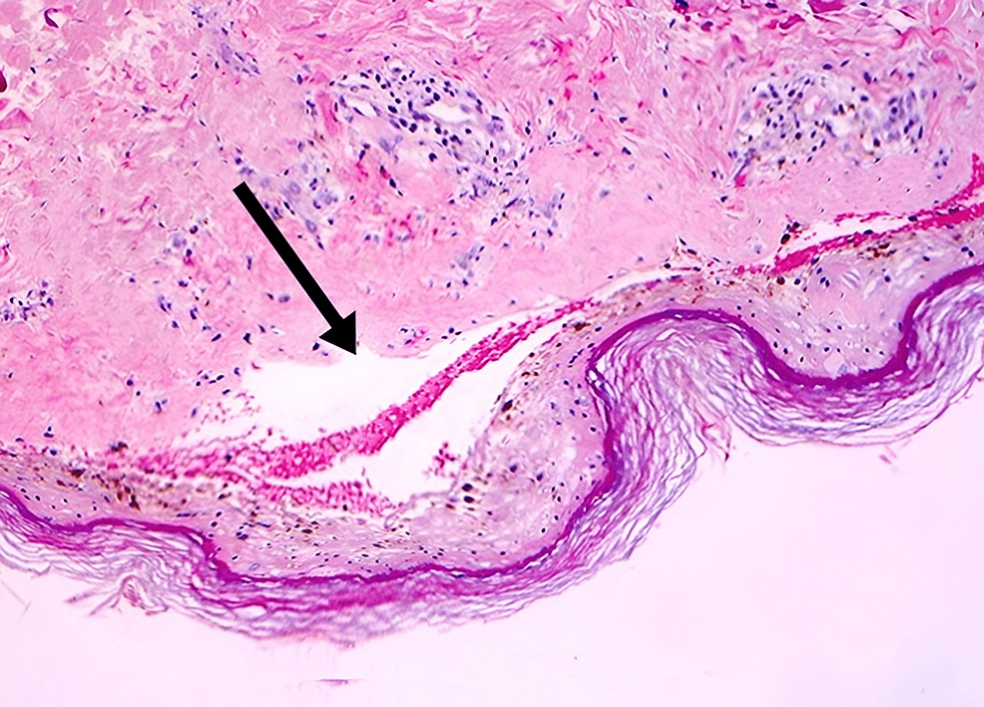
The epidermis eventually is separated or lost due to subepidermal bullous lesions with extensive epidermal necrosis in the histopathology of TEN TEN - toxic epidermal necrolysis

On urine examination, no gram-positive or negative micro-organism was found. No aerobic culture growth was obtained after 72 hours of incubation at 37^o^C. Among differential diagnoses were levofloxacin-induced TEN, scalded skin syndrome, phototoxic skin reaction, and paraneoplastic pemphigus. Histopathology of the skin sample was performed. This investigation was specifically indicated based on the given differential diagnosis. Histopathology of the skin lesion revealed a separated epidermis secondary to a subepidermal bullous lesion (Figure 2).

Upon presentation to the dermatology outpatient department, levofloxacin was discontinued due to its consideration as the primary suspect contributing to the manifestation of toxic epidermal necrolysis (TEN). There was a direct temporal association between the intake of the drug and the start of skin lesions. After discontinuation of levofloxacin, the symptoms started to settle. There were not any new lesions on the skin. It is to be noted that the anti-epileptic drug was not discontinued for this duration.

Based on history, examination, and investigations, levofloxacin-induced toxic epidermal necrolysis was suspected. A definitive diagnosis of TEN was made based on histopathological studies and clinical correlation.

After the establishment of the diagnosis, supportive care was the mainstay of treatment. Withdrawal of possible causative agents and supportive care are the best approaches for the treatment of TEN. The patient was treated in an intensive care unit. Fluid and electrolyte balance, pain management, nutritional support, along with protective dressing were monitored carefully. Parenteral prednisolone 1mg/kg/day was also started.

The patient had improved significantly a week after admission. Liver function tests (LFTs) showed improved liver enzyme status where it was initially deranged. Alanine transaminase (ALT), aspartate aminotransferase (AST), and alkaline phosphatase (ALP) values were 98, 86, and 106 U/L, respectively. The patient was discharged with carefully explained guidelines regarding care of the skin condition. The patient was advised for regular follow-ups at the dermatology clinic for improved management at home.

## Discussion

Lyell first identified toxic epidermal necrolysis (TEN), popularly known as Lyell's disease, in 1956. Necrolysis refers to the necrosis and detachment of the entire thickness of the epidermis, whereas the adjective toxic alludes to constitutional symptoms [5].

Among the differential diagnosis were scalded skin syndrome, toxic shock syndrome, phototoxic skin reaction, and paraneoplastic pemphigus. Thorough history, examination, and array of investigations, including complete blood count (CBC), polymerase chain reaction (PCR) (for serum toxin), urinalysis, and histopathology of skin lesions, helped in ruling out these possibilities.

Drug-induced liver injury was also concerning at the time of presentation of the patient. Differentials for this pathology can include acute viral hepatitis, autoimmune hepatitis, non-alcoholic steatohepatitis, cholecystitis, cholangitis, alcoholic liver disease, or malignancy. Through the investigations explained above, all of these possibilities were ruled out. And drug-induced hepatotoxicity was established as the cause of liver function test derangement. And it was proved by the improvement of liver function tests (LFTs) after the withdrawal of levofloxacin.

An immunological mechanism is thought to be involved in the pathogenesis of TEN. The target cells are largely drug-modified epidermal cells. Different immuno-inflammatory pathways are engaged, and activated CD8 T-lymphocytes play an early role in these pathways. Significantly more soluble interleukin-2 receptors (sIL-2R) are present in blister fluid, which is most likely due to a local down-regulation of an immune-mediated cytotoxic reaction [6]. Under a microscope, eosinophilic epidermal necrosis is present, along with sub-epidermal bullae development. The dermal vessels exhibit endothelial swell but not necrosis or vasculitis. The basal and lower spinous levels of the epidermis have been damaged ultra-structurally, and the lamina densa has developed clefts. Immuno-florescence is always negative.

Sulphonamides, chlormezanone, nonsteroidal anti-inflammatory medications, imidazole antifungals, cephalosporins, anticonvulsants, and allopurinol are the pharmaceuticals most frequently implicated. TEN and Stevens-Johnson syndrome (SJS) are uncommon severe cutaneous adverse reactions (SCAR) caused by fluoroquinolones, which are broad-spectrum bactericidal drugs. The use of fluoroquinolones as the causative agent has been identified in 31 cases of SJS or TEN to date. Of these, just one was brought on by ofloxacin, while 26 were brought on by ciprofloxacin [7]. A few hours to 22 days are typically the interval between taking medication and the beginning of symptoms, with a maximum of 38 days being reported in the case of anti-tuberculosis medications [6].

Fluoroquinolones have a wide range of activities, which makes them quite popular; since they can cause T cell-dependent reactions like SJS and TEN, their benefit-risk profile needs to be carefully examined.

The severity-of-illness scoring system SCORTEN is utilized to evaluate the prognosis and mortality risk of patients with TEN. Elements that SCORTEN considers, include age, malignancy, heart rate, serum blood urea nitrogen, compromised body surface area, serum bicarbonate, and serum glucose. According to this system, one point is assigned to each parameter based on a certain cut-off value, and the total score obtained is used to predict the likelihood of mortality in TEN patients. SCORTEN score can range from zero to seven. A score of three or more represents a bad prognosis with a mortality of up to 35% or higher. In the case being discussed, a SCORTEN score of one was awarded to the patient, which represents a very good prognosis.

Acute tubular necrosis, pulmonary edema, membranous glomerulonephritis, gastrointestinal hemorrhage, bronchopneumonia, and disseminated intravascular coagulation are a few complications associated with TEN. There may also be corneal opacities, cicatricial alopecia, anonychia, ectropion, and entropion. Relapses are common, and recovery takes 14-28 days on average. There may be reticulate skin pigmentation across the impacted areas [8].

The primary goals of treatment are supportive, including removing the precipitating substance, providing excellent nursing care, ideally on a ripple bed, caring for the eyes and mouth to avoid infection and scarring, and maintaining fluid and electrolyte balance. A total of 0.7 ml/kg/% surface area of isotonic saline and 1 ml/kg of body surface area implicated in macromolecules (albumin) should be administered intravenously throughout the course of the first 24 hours. Additionally, 1500 ml of fluids should be administered via the nasogastric tube during the course of the first 24 hours. It is advisable to add potassium to intravenous fluids.

## Conclusions

In conclusion, although being a well-tolerated medication, fluoroquinolone has the potential to cause idiopathic hypersensitivity reactions, such as liver damage or potentially fatal toxic epidermal necrolysis (TEN). As in our situation, early detection and fast treatment of the patient help to avoid fatal outcomes. Lack of oversight of the use of antibiotics and the accessibility of over-the-counter medications in developing nations not only encourages bacterial resistance but also the occurrence of such complications that may go undetected. To stop such occurrences, the government should adopt strong regulations on the prescribing of antibiotics and implement an antibiotic stewardship program at every level of healthcare.

Moreover, this case beautifully manifests Hickam's dictum as well. As the patient was on an anti-epileptic drug for the control of epilepsy, it would have been easier to frame the carbamazepine for TEN. But thorough history, especially medication history, examination, and investigations, uncovered levofloxacin to be the cause. That said, the importance of thorough drug history is also important while dealing with a case of TEN.
